# Utilising simulation to address national workforce and quality priorities: learning from the first four years of the NHS Scotland Academy

**DOI:** 10.1186/s41077-026-00466-3

**Published:** 2026-07-14

**Authors:** Victoria Ruth Tallentire, Clair Elizabeth Graham, Jeanette Karen Stevenson

**Affiliations:** 1 Scottish Centre for Simulation and Human Factors, NHS Forth Valley, Larbert, Scotland; 2 NHS Lothian, Edinburgh, Scotland; 3 Public Services Delivery Scotland, Edinburgh, Scotland; 4 NHS Scotland Academy (Public Services Delivery Scotland), Clydebank, Scotland; 5 NHS Scotland Academy (NHS Golden Jubilee), Clydebank, Scotland

**Keywords:** Health systems improvement, Workforce acceleration, Translational simulation, National simulation programme, Educational governance, Endoscopy training, Anaesthetic practitioner training

## Abstract

NHS Scotland Academy functions as a national mechanism for translating Scottish Government workforce and quality priorities into deployable, simulation-enabled interventions. This article is presented in three parts. The first section describes how NHS Scotland Academy operationalises simulation within a commissioning-to-closure infrastructure comprising staged initiation and approvals, collaborative co-design, structured delivery supported by embedded faculty development, iterative evaluation, proportionate change control, annual review, and formal close/transfer processes. Part 2 presents two exemplar programmes which illustrate how this lifecycle connects simulation to healthcare quality and safety through a translational simulation lens: the Accelerated Anaesthetic Practitioner Programme, commissioned to address prolonged anaesthetic practitioner training and perioperative service pressures; and the National Endoscopy Training Programme, designed as a simulation-powered workforce and quality driver within endoscopy recovery. Building on the broader evolution of IPO (inputs-processes-outputs) in team science, Part 3 suggests the ways in which the learning from NHS Scotland Academy may contribute to the development of translational simulation theory, particularly through recognition of mediating mechanisms, episodic temporality, and cross-level causation. NHS Scotland Academy’s infrastructure and exemplars make these features more visible and suggest how the theory might be refined while retaining the pragmatic utility of IPO. Overall, this Advancing Simulation Practice article positions NHS Scotland Academy both as an effective national delivery platform for simulation-enabled workforce and quality programmes and as a testbed for advancing translational simulation conceptual models.

**Graphical Abstract:**

**Figure:**
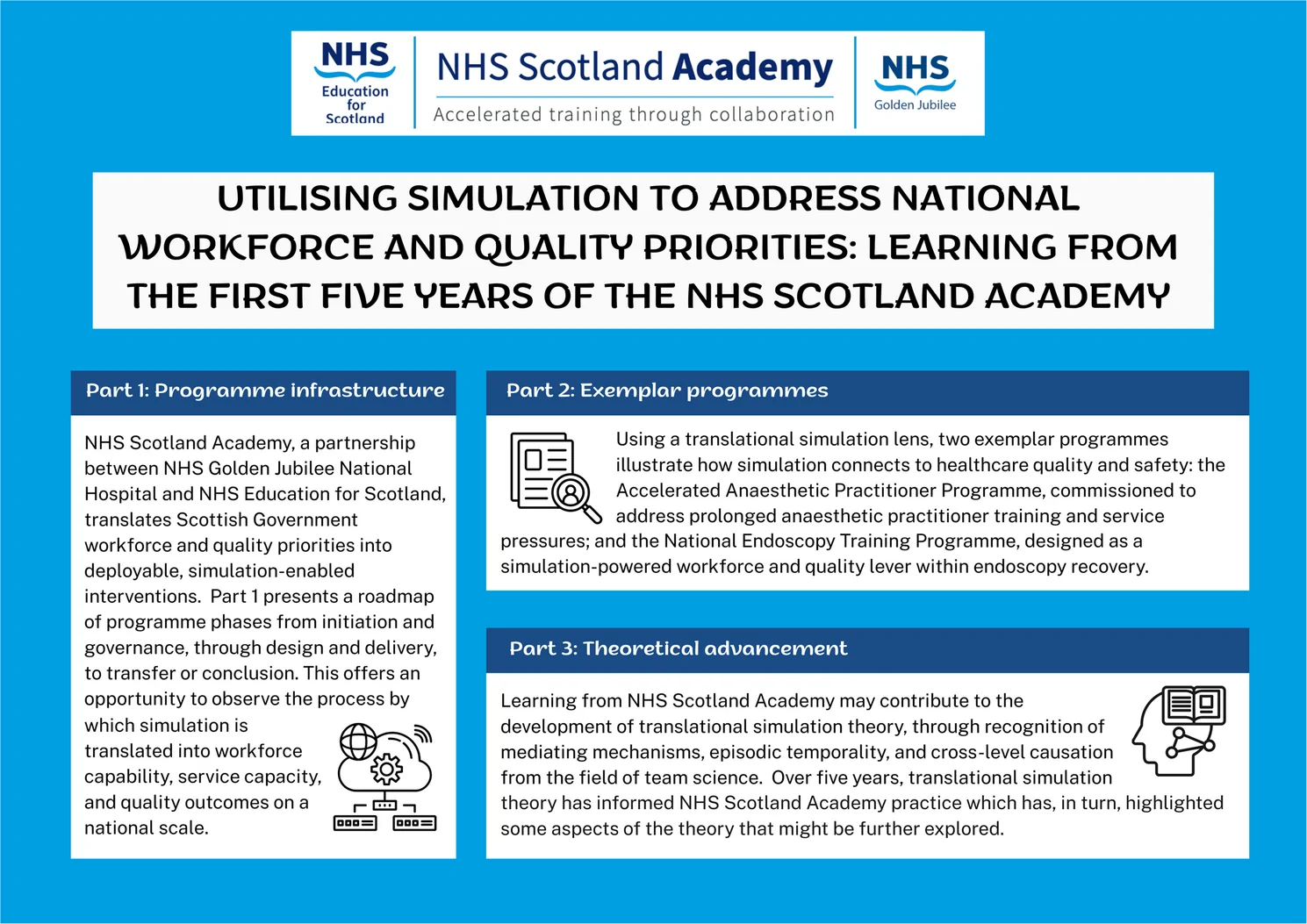

**Supplementary Information:**

The online version contains supplementary material available at https://doi.org/10.1186/s41077-026-00466-3.

## Background

A persistent challenge in the simulation field is that high-quality simulation frequently remains structurally peripheral, reliant on local enthusiasm and fragile resourcing, with limited mechanisms to scale, sustain and align activity with service outcomes. Over the last four years, the NHS Scotland Academy (NHSSA) has done things differently; offering novel programmes that make simulation actionable at scale, operationalising it as part of wider intervention ‘bundles’ that integrate immersive supervised practice, technology-enabled delivery, faculty development and evaluation. Established in October 2021, NHSSA is a partnership between NHS Golden Jubilee University National Hospital and the organisation previously known as NHS Education for Scotland (NES), now part of Public Services Delivery (PSD) Scotland [1]. NHSSA was designed to deliver a national accelerated approach to workforce and quality priorities while avoiding duplication: it fills gaps, convenes partners to tackle complex problems, fosters collaborative design, creates content and provides training. To make the scope of NHSSA’s work more tangible, Table 1 summarises the range of programmes and resources developed or supported by NHSSA alongside the workforce or quality priorities they were designed to address.

**Table 1:** NHSSA programmes and the workforce or quality priorities they address

Workforce / quality priority	Programme
**Current programmes**	
Improve endoscopy quality, reduce waiting times and support delivery of care to national standards	National Endoscopy Training Programme
Expand and develop the endoscopy workforce by supporting Health Care Support Workers to progress to Assistant Endoscopy Practitioner roles	National Assistant Practitioner (Endoscopy) Programme
Support registered nurses new to endoscopy practice to gain the knowledge, skills and confidence required for safe endoscopy care	Registered Nurse / Foundations of Endoscopy Practice Programme
Increase surgical workforce capacity and capability by developing experienced perioperative practitioners into Surgical First Assistants	Surgical First Assistants Programme
Accelerate completion of anaesthetic practitioner competencies, particularly high-impact, low-exposure skills, to support elective surgical capacity	Anaesthetic Practitioner Programme
Support registered nurses who are new or returning to perioperative practice to develop the core knowledge and skills required for safe perioperative care	Foundations of Perioperative Practice Programme
Enable Health Care Support Workers in perioperative environments to progress to Assistant Perioperative Practitioner roles and contribute to surgical delivery	National Assistant Perioperative Practitioner Programme
Strengthen the Central Decontamination Unit workforce by providing accelerated education for decontamination technicians	National Decontamination Science / Central Decontamination Unit Workforce Training Programme
Support implementation of the High-Volume Cataract Surgery blueprint through digital learning resources for specific perioperative cataract pathway skills	High-Volume Cataract Surgery Resources
Increase cataract surgical training capacity through immersive placements for senior residents in high-volume cataract surgery settings	Cataract Surgery Immersive Training Programme
Equip independent prescribing pharmacists with clinical skills and confidence to begin prescribing, thereby improving access to care and reducing pressure on General Practice services	National Clinical Skills for Pharmacists’ Programme
Increase ultrasound capacity by supporting NHS Boards to train ultrasonographers and doctors in general medical and selected specialist ultrasound	National Ultrasound Training Programme
Improve bronchoscopy capability and capacity, including basic bronchoscopy and endobronchial ultrasound-guided procedures relevant to lung cancer staging	National Bronchoscopy Training Programme
Increase primary and acute care ear-care capacity by training registered nurses to become trainers in micro-suctioning	Ear Care Programme
**Completed programmes now passed to other providers as business-as-usual**	
Support NHS Scotland Boards recruiting internationally educated nurses and midwives by helping candidates and educators prepare for Nursing and Midwifery Council exam requirements	Nursing and Midwifery Council OSCE Preparation Programme
Promote culturally humble values and behaviours across health and social care, supporting inclusion, staff wellbeing and person-centred care	Cultural Humility Resource
Support recruitment and induction into health and social care roles through a national pre-induction digital learning resource	Preparing for Work in Health and Social Care in Scotland
Explore approaches to widening access and future workforce pipeline development through school-age engagement with NHS careers	Youth Academy Pathway Pilot
Accelerate biomedical science registration by supporting graduates to complete practical elements of the Institute of Biomedical Science Registration Training Portfolio	Accelerated Institute of Biomedical Science Registration Training Portfolio Programme

Furthermore, the features that make NHSSA an effective delivery platform for programmes also create the conditions for a real-world testbed in which commissioning, implementation, governance and measurement are treated as integral to simulation practice. Likewise, simulation is viewed as a key part of toolkit utilised to address national, systemic workforce and quality priorities. This infrastructure offers simulation educators and researchers an opportunity to observe how simulation is translated into workforce capability, service capacity and quality outcomes on a national scale. In Part 1 of this Advancing Simulation Practice article, we describe how simulation has become embedded as part of NHSSA’s infrastructure, outlining the commissioning, governance and delivery processes. Subsequently, in Part 2, we illustrate how this approach connects simulation with healthcare quality and safety through two detailed exemplars. Part 3 concludes by exploring the ways in which the first four years of experience at NHSSA may inform current theoretical models of simulation activity focussed primarily on improving patient care and healthcare systems.

### PART 1 - description of practice: NHSSA infrastructure

**Fig. 1:**
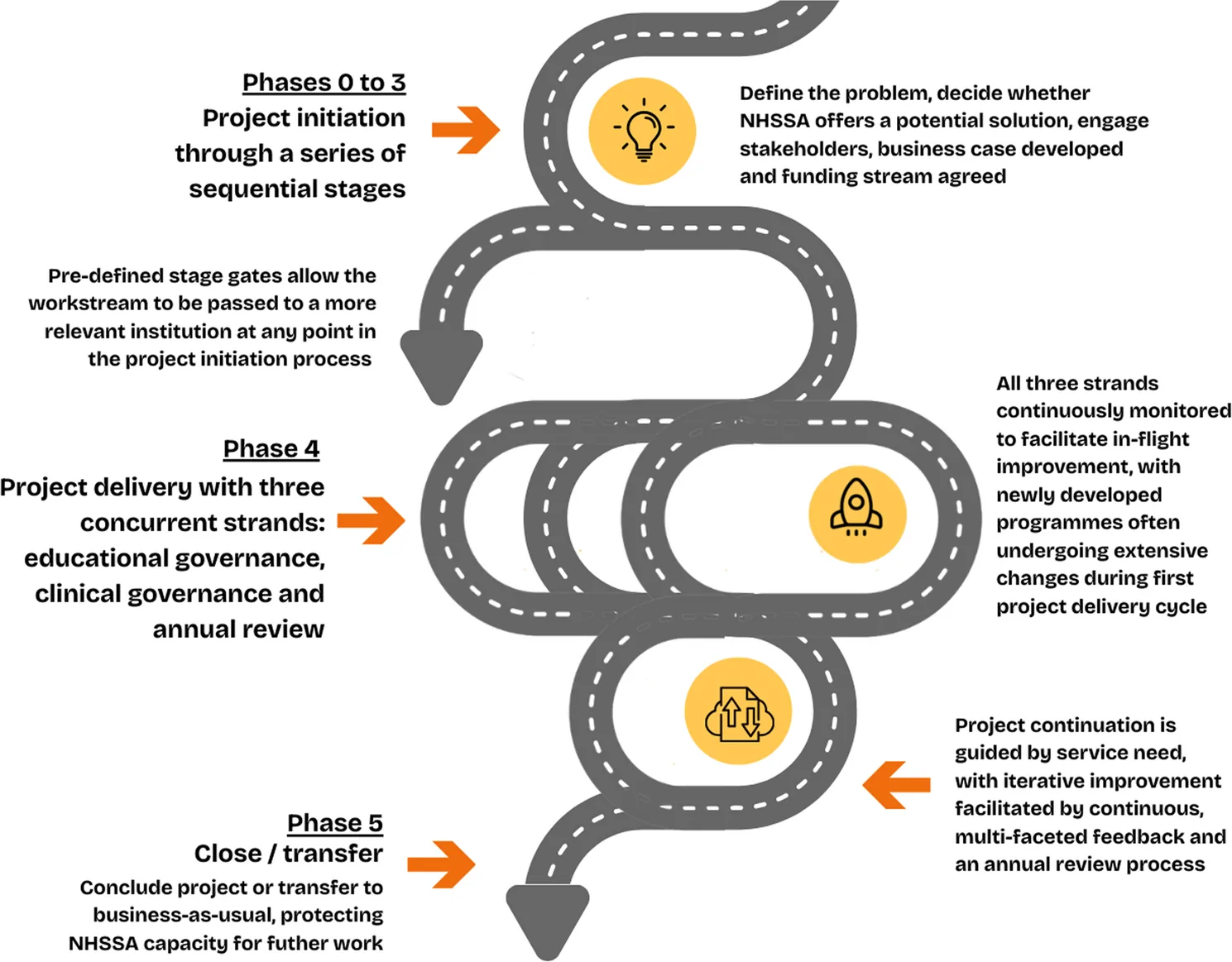
Roadmap of the NHSSA project lifecycle phases, from project initiation to pre-delivery planning, delivery, and eventual conclusion or transfer. The pathway is shown sequentially to reflect required stage dependencies; however, programme development is iterative within and between stages, with stakeholder feedback, governance review, change control and annual programme review informing adaptation throughout

NHSSA operates as a conduit that translates nationally prioritised workforce and quality needs into deployable simulation interventions. This section focuses on how the system works in practice, detailing the lifecycle and governance of programmes. At a high level, NHSSA activity moves through the key phases shown in Fig. 1. Although the broad phases are shown sequentially because initiation, governance, planning and faculty development are necessary prerequisites for delivery, iteration occurs within and between phases through stakeholder feedback, governance review, change control and annual programme review.

#### Project initiation

NHSSA supports the exploration of complex or systemic issues affecting the health and social care workforce, as identified at Scottish Government (SG) level. Between seven and ten workstreams typically sit within the project initiation phase at any time. Exploration and initiation follow defined stages involving discussion of the initial idea with NHSSA and SG sponsors; initial paper submission to the Clinical Advisory Group; Executive Programme Group (EPG) approval; and subsequent further rounds of approvals of the detailed business case by both oversight groups, with funding arrangements agreed. The scale and funding arrangements for NHSSA programmes vary according to the nature of the workforce or quality priority being addressed. NHSSA receives SG funding, including a recurring allocation to support the core programme team and additional allocations for specific workforce-priority workstreams. Individual projects may be funded by SG policy teams, the Centre for Sustainable Delivery or, in some cases, partner organisations. Financial oversight is maintained through NHSSA governance processes, with financial summaries forming part of project close documentation. The project initiation phase emphasises collaboration with stakeholders across health and social care, tertiary education, regulators and professional bodies. At each stage, the key judgement is whether the issue represents a national workforce or quality priority for which an accelerated, collaborative NHSSA response is appropriate. If an accelerated NHSSA solution is deemed not to be appropriate, pre-defined stage gates allow the workstream to be passed to a more relevant body or partner institution at any point in the project initiation process.

Once clear and credible specification of programme needs and the expectations of commissions have been established, NHSSA uses the NHS Scotland Academy Learning Design Roadmap (SALDR), adapted from similar roadmaps [2, 3] based on Carpe Diem learning design [4]. SALDR is a two-session collaborative team-based development workshop that ensures an appropriate mix of representatives within the design process, along with integration of quality management and continuous feedback processes. When a potential NHSSA workstream has been identified and judged to align with a national workforce or quality priority, the next task is to determine whether the programme is ready to move from concept to deliverable intervention. In NHSSA, this readiness judgement is made through three interdependent forms of governance: clinical, educational and operational.

#### Governance and readiness

For NHSSA, governance is not a separate administrative layer but a central mechanism through which national workforce or quality priorities are converted into deliverable educational programmes. NHSSA programmes frequently cut across professional groups, territorial Health Boards, national policy teams, education providers and service-delivery structures, meaning that programme readiness depends on more than curriculum design. It requires clarity about the problem being addressed, legitimacy to act nationally and operational permission to proceed. Three non-sequential strands form the governance and readiness phase: educational governance; clinical governance; and faculty recruitment and development. Clinical governance sits within the relevant territorial Health Board, whilst educational governance is managed through NHSSA’s EPG, with regular scrutiny of activity creating a governance rhythm that supports oversight whilst enabling pace.

Faculty are often recruited directly from clinical practice settings, support by a structured faculty development programme covering both theoretical and practical aspects. The simulation-related parts of the faculty development programme were developed in collaboration with the Scottish Centre for Simulation and Clinical Human Factors, and cover simulation design, delivery and debriefing [5]. Together, these governance dimensions function as a readiness check and an implementation scaffold determining whether a proposed intervention is appropriate for NHSSA, how it should be designed and what conditions need to be in place before delivery can begin.

#### Delivery

Delivery of programmes commences when all three governance and readiness strands are adequately established and agreed. During delivery, feedback functions as a continuous quality mechanism rather than a retrospective evaluation exercise. Feedback is gathered from multiple sources, including learners, faculty, workplace supervisors, programme teams and, where relevant, service stakeholders. This may include post-session evaluations, informal faculty debriefings, learner progress data, supervisor reports, assessment outcomes and issues raised through programme governance meetings. Programme teams review these data iteratively, distinguishing between minor delivery adjustments that can be made rapidly, curriculum or assessment changes requiring educational governance review, and wider operational issues requiring escalation to strategic or clinical governance groups. Changes are logged through programme documentation and incorporated into annual programme review or project-close reports. In this way, feedback contributes both to local programme refinement and to NHSSA-wide quality assurance by making visible what is working, what requires adaptation and what needs to be communicated to commissioners, Health Boards, partner organisations or faculty groups. From June 2024, NHSSA has used a defined process for project change to manage adaptations without losing control of scope, cost or governance. Small changes or extensions to programmes (with an estimated cost of less than £15,000) are distinguished from larger changes or extensions, ensuring proportionate escalation and transparency for sponsors and governance groups.

#### Conclude or transfer

NHSSA has a formal project conclusion or transfer phase, again overseen by the EPG. In practice, transfer begins with a formal close-and-evaluation review, drawing together the benefits log, change log, lessons learnt, annual review data, stakeholder feedback and financial summary. These materials are used to determine whether the original commission has been met, whether there remains an ongoing need for the programme, and where future ownership should sit. Where ongoing maintenance is desirable, NHSSA may transfer a programme to PSD Scotland or another public body as part of their business-as-usual offer. At that point, responsibility for maintenance, content currency, stakeholder relationships and further development transfers to the receiving organisation; no funding accompanies the transfer, and any transfer of NHSSA-generated intellectual property requires approval through NHSSA governance. Importantly, NHSSA’s close or transfer process is explicitly framed as a capacity-creation mechanism: projects are closed after the initial commissions are met to create capacity for new areas of workforce challenge and emerging priorities.

This transition phase is therefore not simply an administrative endpoint, but a core part of the NHSSA model. It creates an explicit mechanism for moving successful resources or programmes from accelerated development into sustainable ownership, while releasing NHSSA capacity to respond to new workforce and quality priorities. Early examples illustrate the anticipated pattern, for example the Cultural Humility resource included in Table 1 has recently been handed over to the PSD Scotland Equality, Diversity and Human Rights Team as part of business-as-usual.

### PART 2 - from lifecycle to exemplars: putting processes into practice

Having outlined NHSSA’s commissioning-to-closure lifecycle, we now build on the model of how work is commissioned, governed and delivered to demonstrate how it functions in practice through two exemplars. To structure these exemplars, we have viewed the process roadmap detailed above through a translational simulation (TS) lens. TS is a way of framing and organising simulation activity such that its primary purpose is to directly improve patient care and healthcare systems – not just train individuals or teams [6]. Over the last decade, TS has evolved from a functional descriptor [6, 7] to a practical operational model incorporating input-process-output (IPO) thinking from the field of organisational psychology [8, 9]. Subsequent work has emphasised conceptual refinement and quality criteria, advocating clear purpose that explicitly connects to the simulation process [10]. Most recently, TS has been championed as part of a wider improvement system embedded within quality and safety communities and governance structures [11].

NHSSA’s programmes developed and matured in parallel with wider academic discourse on TS, and the principles heavily influenced how NHSSA programmes have utilised simulation to address service-facing problems, whereby improvements in capability, reliability and team performance translate into quality and safety outcomes. In the two exemplars described, the proximal mechanisms are recognisably educational. However, the programme logic in each case is translational, whereby simulation is used within a governed national intervention designed to address workforce capacity, service readiness and quality priorities. Nickson and colleagues’ IPO model resonates with the NHSSA programmes through an emphasis on how simulation should be treated as a health-service improvement intervention, deliberately linked to governance, implementation and evaluation, rather than a standalone training event [8]. In the NHSSA exemplars, this connection was operationalised by ensuring that simulation activities were not designed or evaluated solely within educational teams, but were linked to programme governance, stakeholder review, workforce priorities and external accreditation structures. Feedback was therefore able to inform programme modification, escalation through governance routes and, where appropriate, alignment with wider organisational processes. In this sense, feedback from the programmes generated organisational learning about the conditions required for workforce development to produce service-level impact. In the IPO model, *inputs* clarify the service problem, stakeholders, context and measures; *processes* are the simulation activities designed to diagnose, test change or address a service need; and *outputs* are the actionable system changes and feedback pathways [8].

#### Exemplar 1: the accelerated anaesthetic practitioner programme

In Scotland, an anaesthetic practitioner is a perioperative clinician (typically a registered nurse following completion of the PSD Scotland Core Competency Framework) who works alongside an anaesthetist to deliver care through the anaesthetic part of the patient journey. In practice, they assist from induction of anaesthesia through to immediate post-theatre recovery, acting as the anaesthetist’s named assistant for the list: preparing and checking specialist equipment and drugs, supporting airway management and monitoring, anticipating complications, and helping coordinate the team so that anaesthesia is delivered safely and reliably.

#### Project initiation

The Accelerated Anaesthetic Practitioner Programme (AAPP) was commissioned in August 2022, in response to Scottish Health Boards’ reports that timely completion of the relevant competency framework had become increasingly challenging due to service pressures and variable access to ‘high-risk, low-exposure’ clinical experiences (e.g. emergencies, advanced airway management, paediatrics, obstetrics, and remote working). The resulting prolongation of training (beyond the recommended 6–12 months, to a median of 20 months with a range of 12–36 months) had created a shortage of trained anaesthetic practitioners and was contributing to increased patient waiting times for surgery.

#### Governance and readiness

The programme’s design uses a hybrid accelerated learning model of structured digital preparation with eight face-to-face workshops over a 26-week period. The workshops are complemented by high-fidelity, fully debriefed simulated scenarios targeted directly at the rate-limiting competencies, such as rapid sequence induction and patient transfer. The online, workshop, and simulation components are intended to complement and enhance workplace learning, reduce reliance on opportunistic exposure, and support more timely and confident progression to competent practice. The programme is delivered through collaboration between anaesthetic clinical experts, simulation educators, NHSSA programme staff, workplace supervisors and employing Health Boards. See Supplementary Table 1 for additional contextual details.

#### Delivery

In terms of scale, the first three and a half years of the programme have involved eight cohorts of learners, with 70 individuals in total. Sixty-seven (96%) learners completed all required competences, with two leaving the course due to resignation from their practice roles and one paused due to long-term sick leave. In the AAPP exemplar, feedback has operated at several levels. Learner and faculty feedback informed iterative changes to workshop design, sequencing and supporting resources. Workplace feedback from supervisors and Health Boards helped identify where learners required additional support to translate workshop learning into clinical practice and competency completion. Programme-level review then allowed recurring issues to be considered alongside cohort progression, enabling the programme team to distinguish between changes that could be made immediately within the next delivery cycle and issues requiring broader discussion, such as faculty sustainability and professional recognition. Feedback therefore functioned as an operational mechanism to inform iterative improvement of the programme while maintaining alignment with the original workforce priority.

Consistent with TS principles that outputs should align with organisational reporting cycles and governance, progress is reported in an annual programme review that consolidates performance metrics (e.g. cohort completion and competency completion), stakeholder feedback, and changes made during the review period [8]. As a result of the programme, there has been a significant impact on service provision in terms of increased workforce capability, flexibility, and resilience, as reported by service managers. Formal dissemination of impact has begun through presentation at national simulation meetings [12], with ongoing intent to share methods and outcomes beyond the local setting [8]. Outputs are used to adapt the programme and its implementation conditions (e.g. changes to cohort scheduling to reduce winter disruption and service pressure, and plans for faculty sustainability and potential regional delivery models), according with the TS principle of simulation embedded as part of a strategy, not an isolated event, with iterative recalibration of inputs and processes. The conclusion or transfer phase of the programme has not yet been reached.

**Fig. 2:**
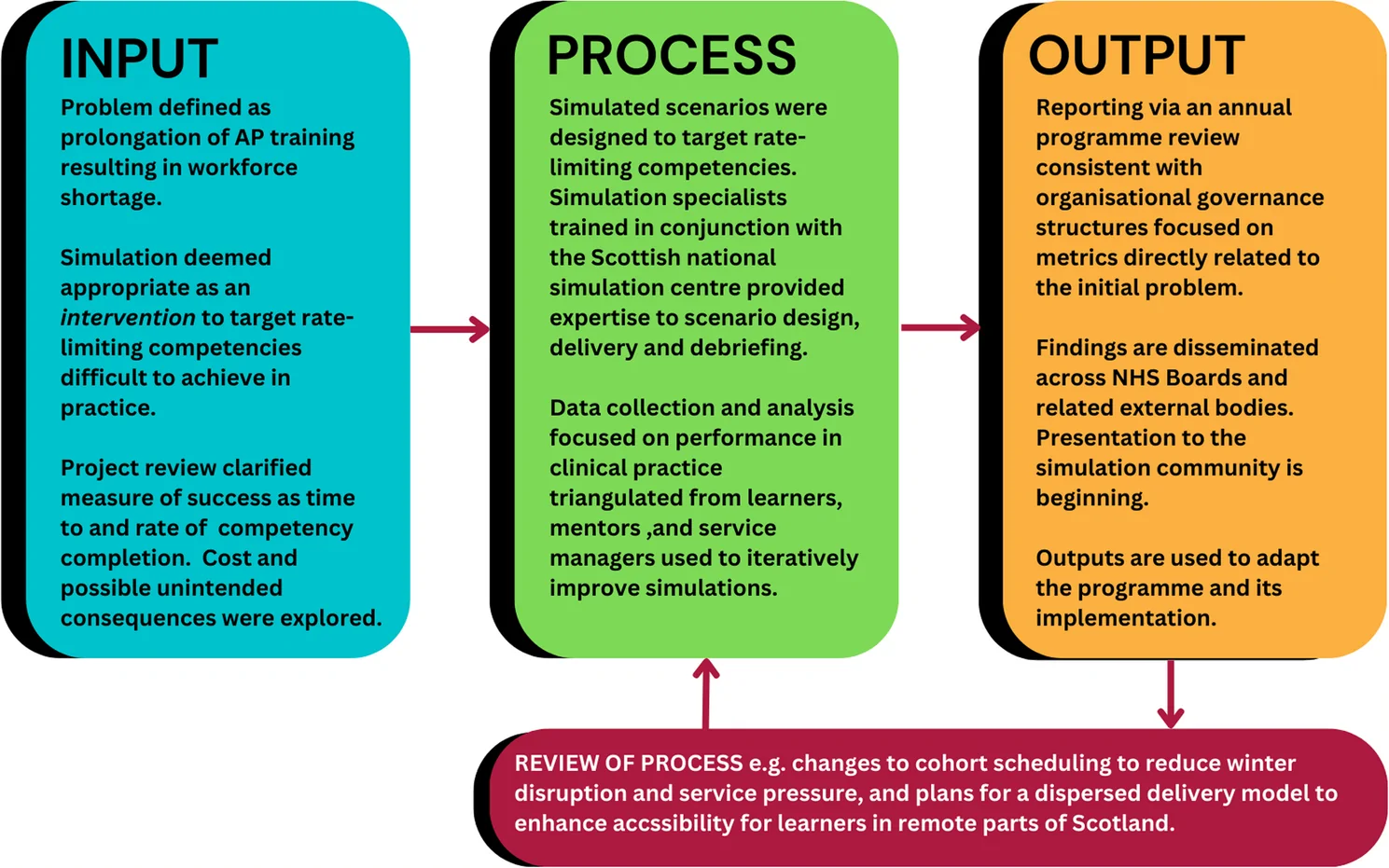
The accelerated anaesthetic practitioner programme using an IPO model

Whilst the AAPP utilises simulation primarily as a tool for accelerated education, it is embedded within a framework addressing service-level needs. The TS contribution of AAPP therefore lies in the way simulation activities were organised as part of a national response to anaesthetic practitioner workforce constraints and elective surgical capacity. The clear through-line from the *input* problem (capacity and risk in perioperative workforce development), through the *process* of simulation-enabled intervention design and delivery, to the *outputs* that matter in quality and safety terms is shown in Fig. 2.

#### Exemplar 2: the national endoscopy training programme

Endoscopy refers to the visual examination of internal luminal structures using an endoscope - a flexible or rigid instrument equipped with an illumination source and video system, often with working channels to enable diagnostic sampling and therapeutic intervention (e.g. polypectomy, haemostasis, dilatation). In Scotland, the term endoscopy encompasses three key procedures: upper gastrointestinal endoscopy, colonoscopy and flexible sigmoidoscopy.

#### Project initiation

Prior to the COVID-19 pandemic, endoscopy had been recognised as an area needing ‘substantial, sustainable change’, prompting a SG action plan published in March 2019 [13]. The pandemic amplified an existing fragility: endoscopy services were heavily disrupted by infection-control constraints and redeployment, and the system accumulated a large diagnostic backlog. In March 2021, 33,444 patients in Scotland were waiting for an endoscopy and by June 2021, delivery of new patient endoscopy procedures within six weeks had fallen, from 66.6% a year earlier, to 36.5% [14]. In mid-2021, the SG Health and Social Care Management Board approved the initial concept of the National Endoscopy Training Programme (NETP), developed as a simulation-powered workforce and quality lever within a wider recovery agenda, with the explicit aim of expanding training capacity and quality infrastructure to unlock service capacity.

#### Governance and readiness

The design process began with a clear articulation of the service problem, its scale, key stakeholders and success measures, so that simulation was co-designed for health-service outcomes rather than education in isolation [8]. NETP was framed around national service needs including capacity to address waiting times; a sustainable skilled workforce; avoidance of repeat procedures; and improvement patient outcomes and experience. Against the prevailing service pressure, NETP’s stated inputs also included enhancing the quality of endoscopy through gaining Joint Advisory Group for Gastrointestinal Endoscopy (JAG) clinical and training accreditation [15].

#### Delivery

NETP operationalised these aims through a hub-and-spoke national training model, with the central hub hosted at NHS Golden Jubilee University National Hospital (including a dedicated national training centre with procedure rooms and teaching space) and regional delivery expanding as capacity and accreditation developed. JAG-supported, standardised and accredited courses (rather than ad hoc local training) were spread across multiple Scottish sites. Embedded within this strategy, simulation was used in complementary ways. Hands on ‘model work’, with individualised learning plans, was used to build core scope handling skills alongside supervised clinical immersion. Following this, virtual-reality (VR) endoscopy simulators, provided in each training region of Scotland, helped learners accelerate early acquisition of endoscopy skills and practise insertion and withdrawal, tip control, basic skills, and pathology recognition in a supervised simulation environment. In NETP, simulation activity was linked to external organisational processes through alignment with JAG standards, national trainer-development activity and regional training-centre development. Feedback from learners, faculty and endoscopy services informed wider programme decisions about accreditation support, training capacity, standardisation of practice and the development of additional workstreams. See Supplementary Table 1 for additional contextual details.

Following the first four years of the programme, clear signals of decreased waiting times and increased quality of care exist. In terms of scale, over 200 endoscopists have now completed colonoscopy training though NETP, and over 1,500 patients are estimated to have avoided a second colonoscopy through improved procedure completion rates [16]. National data shows a 14% increase in detection rates for polyps – small growths that can develop into cancer – and all NHS Scotland Boards are now reaching national standards for colonoscopy procedures [17]. At the wider system level (not attributable to NETP alone), Public Health Scotland diagnostics statistics show endoscopy waiting-list size decreased from 33,162 (at end December 2024) to 32,956 (at end March 2025), then to 30,787 endoscopy waits at end Sept 2025, and most recently to 29,661 at end December 2025. The latest figure represents a further 4% quarter-on-quarter reduction and an overall reduction of approximately 11% from December 2024 to December 2025, consistent with wider service recovery rather than an effect attributable to any single programme [18]. Having initially focussed on technical skills development, the importance of non-technical or behavioural skills on the quality and safety of endoscopy was increasingly recognised [15], resulting in its incorporation within more recent iterations of the programme [19]. In addition, a ‘training the colonoscopy trainers’ course has been developed and delivered to 60 colonoscopists. The conclusion or transfer phase of the programme has not yet been reached.

**Fig. 3:**
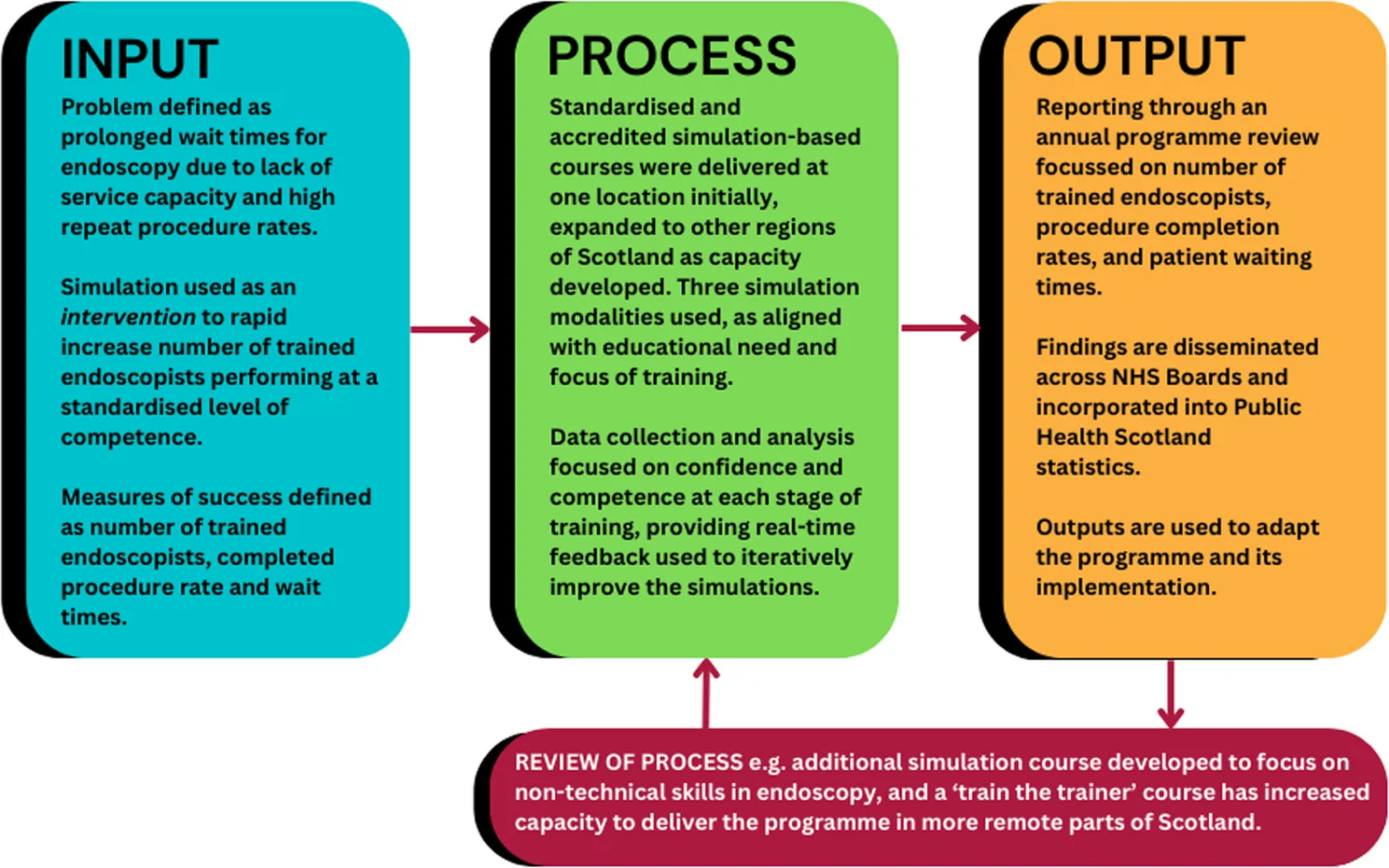
The national endoscopy training programme using an IPO model

Like AAPP, NETP utilises simulation primarily as a tool to provide enhanced, accessible and timely education. However, its TS contribution lies in the way this education is embedded within a national strategy deliberately designed to address service-level priorities of improving endoscopy quality, expanding training capacity, supporting accreditation and reducing variation in care delivery. Figure 3 delineates the *input* problem (increasing service capacity through improved training and quality infrastructure), the *process* of simulation-based education through a hub-and-spoke national training model, and the *outputs* in terms of waiting times and quality of care.

### PART 3: using practice to inform theoretical advancement

Although the two exemplars include simulation activities directed largely at individual learner capability, they also demonstrate that the translational work surrounding those activities is inherently collective. In both cases, simulation could only contribute to workforce or quality outcomes because multiple groups aligned their work around a shared service-level purpose. Nickson and colleagues’ adaptation of an IPO model for TS evolves from the team effectiveness and quality management traditions [8]. In the wider organisational and social psychology literature, IPO is commonly traced to McGrath’s work on group effectiveness, in which antecedent conditions (inputs) shape interactional activity (processes), which in turn generate valued outcomes [9, 20]. Both Nickson et al.’s model, and Brazil’s more recent paper promoting a data strategy across input, process and output phases that is explicit and disciplined, re-specify the IPO model for healthcare improvement, as described above in Part 2 [8, 21]. Building on this advancement of TS framing, we have contextualised our work within the conceptual evolution of IPO seen in team science. This approach resonates with both our personal experience of TS as a ‘team sport’ and the emerging consensus view of teams as complex, adaptive, dynamic systems [22]. Across the wider literature, IPO models have been progressively refined to address three recurrent limitations: over-broad use of the term ‘process’, insufficient representation of phases or episodes, and under-specification of context and multilevel effects [20, 23, 24]. Marks et al. distinguished team processes as time-sensitive and episodic (e.g. transition, action and interpersonal processes), while Ilgen et al. argued that many variables treated as ‘processes’ are better conceptualised as mediators, including affective, cognitive and behavioural states (e.g. trust, cohesion, shared mental models), leading to the IMOI formulation (input–mediator–output–input) [23, 24]. Mathieu et al. further emphasised the multilevel, contextual and time-sensitive nature of team effectiveness research [20]. In contrast, the TS literature to date remains primarily an operational and practice-organising framework, with comparatively less formal treatment of mediating mechanisms, temporality and cross-level causation. These three aspects may be best explained using aspects of the NHSSA programme lifecycle detailed in Part 1 and the example programmes described above in Part 2.

### How NHSSA experience helps to advance understanding

(i) Mediating mechanisms

The notion of *mediating mechanisms* stems from the recognition that not all factors transmitting the influence of inputs to outcomes are processes; many are cognitive, relational or affective states [23]. The NHSSA infrastructure described above provides an example of why this extension is important. The phases of the NHSSA lifecycle do much more than sequence work: they create conditions that mediate programme success. For readers wishing to identify equivalent mediators in their own TS models, useful questions might include: what builds legitimacy for the work; what creates shared ownership; what develops delivery capability; what allows adaptation without loss of fidelity; and what supports sustainability beyond the initial intervention? In the NHSSA exemplars described in Part 2, potential mediators include governance legitimacy, stakeholder alignment, trust, shared problem definition, collaborative educational design and accreditation alignment. These are not inputs in a static sense, nor are they simple processes; they function as mediating mechanisms because they shape whether simulation outputs can translate into service change.

Making such mediators explicit may also facilitate less linear reporting of TS. Mediators do not always exert a positive effect, and the NHSSA experience suggests that they may also explain delay or attenuation of impact. For example, national stakeholder coordination can create legitimacy and reduce duplication but may also increase complexity and slow decision-making. Similarly, faculty development can build delivery capability, but sustainability becomes vulnerable where specialist faculty capacity is limited. Linking to recent calls for explicit data strategies [21], data infrastructures can support evaluation, but incomplete or inconsistent source data may limit causal claims and jeopardise reinvestment. Reporting these mediating mechanisms, including where they constrain as well as enable impact, may strengthen causal explanation and improve transferability of lessons to other systems.

(ii) Episodic temporality

The *episodic temporality* described in IPO team science literature, where different phases of work produce different classes of outputs, promotes the notion that a project should not be judged by any outcome metric too early [24]. At the intersection of healthcare simulation and system improvement, where those in authority are often under significant pressure to demonstrate rapid improvement from additional investment, this concept gains particular significance. Recent work has moved away from static perspectives of effectiveness and attempted to integrate the effects of temporal processes such as project deadlines and schedule synchronisation [24, 25]. The simultaneous pursuit of multiple goals further complicates the process, nicely illustrated by the AAPP described in Part 2. This programme is not a single intervention but an evolving sequence: commissioning in response to a workforce bottleneck (prolonged anaesthetic practitioner training and service pressure), structured hybrid delivery over 26 weeks, embedded data collection during delivery, annual review, and iterative adaptation of various aspects. The evaluative outputs are likewise time-sensitive, with early outputs including learner confidence and competency progression, while later outputs include workforce capability, flexibility and resilience, with implications for perioperative service provision. The sequencing and progressive nature of these outcomes is significant: over-emphasis on immediate service provision could easily have resulted in a shortened educational programme developing practitioners less able to function in high acuity clinical situations and less adept at moving flexibly between roles as required by service needs. This complexity creates an important tension for TS as programmes such as AAPP and NETP rarely pursue a single educational goal. However, the response is not to reduce ambition or scope, but to maintain disciplined focus at the level of each programme component such that the pressure of overall mission goals is tempered by processes that emphasise quality standards (such as alignment with accreditation standards) and sustainability (such as faculty capacity and operational oversight).

(iii) Cross-level causation

In contemporary team science literature, *cross-level causation* recognises that effects travel between individual, team, unit, organisational and system levels. This idea originates from social science levels-of-analysis inference errors stemming from the misplaced assumption that effects observed at one level (e.g. individuals) automatically hold at another (e.g. departments or services) [26]. Whilst is difficult to represent within TS programmes, the concept of ‘performance composites’ is fundamental to integration with wider improvement initiatives within organisations that are inherently multilevel systems [20, 27]. NETP illustrates this complexity well: its inputs include a national service backlog, a capacity problem and concerns about service quality; its processes span simulation modalities (model work, VR), a hub-and-spoke training infrastructure, and JAG-supported standardisation; and its outputs include individual skill development, trainer development, quality indicators (e.g. polyp detection), and system-level signals such as waiting-list reduction. However, wider waiting-list recovery is multi-factorial and not attributable to NETP alone. It is that type of cross-level causal claim (and the limits of such claims), that might move TS conceptualisation forward through description of multilevel, intersecting pathways of improvement that connect simulation activity to service outcomes. In this way, TS can become embedded within wider organisational improvement initiatives, seen as a central cog that can transmit and amplify the work of other sectors of the organisation.

### Summary

Across its first four years, NHSSA has functioned as a national mechanism for translating SG workforce and quality priorities into deployable, simulation-enabled interventions. NHSSA’s contribution is as much in its infrastructure as its outcomes: simulation is operationalised within a commissioning-to-closure lifecycle that includes defined initiation stage gates, collaborative co-design, structured delivery with embedded faculty development, iterative evaluation, and explicit close or transfer processes designed to protect capacity and enable portfolio renewal. This governance rhythm has allowed NHSSA to develop the agility to implement programmes at pace when required, whilst retaining oversight of activity and fostering the trust of key partners.

NHSSA’s programmes have matured in parallel with, and been significantly influenced by, TS academic discourse. The AAPP and NETP exemplars show how NHSSA aligns with TS-IPO logic: *inputs* are framed as service-facing problems, stakeholder constellations, context and measures; *processes* comprise simulation-enabled design and delivery modalities; and *outputs* are defined in terms of actionable changes, workforce capability, quality signals, and governance-aligned feedback pathways. Importantly, both exemplars illustrate outputs as aspects of performance and adaptation (e.g. AAPP cohort scheduling and sustainability planning; NETP evolving to include behavioural skills and trainer development), reinforcing TS as a strategy enacted through iterative programme cycles rather than a single event.

Based on the wider evolution of IPO in team science, the experiences of NHSSA helps make visible some of the aspects of TS theory that might be further explored, specifically (i) the *mediating mechanisms* that plausibly transmit effects from simulation activity to service change; (ii) the significance of *episodic temporality* to avoid the impact of simulation activity being judged prematurely by distal service outcomes; and (iii) *cross-level causation*, in which effects propagate across individual, team, service and system levels. NHSSA provides both an example of how simulation can be utilised to address national, systemic workforce and quality priorities whilst also providing a platform for advancing TS thinking. But most of all, this is an opportunity to celebrate the first four years of NHSSA, and solidify ambitions for the next decade.

## Supplementary Information

Supplementary Material 1.

## Data Availability

No datasets were generated or analysed during the current study.

## Abbreviations

NHS: National Health Service
NHSSA: National Health Service Scotland Academy
NES: National Health Service Education for Scotland
PSD Scotland: Public Services Delivery Scotland
SG: Scottish Government
EPG: Executive Programme Group
SALDR: NHS Scotland Academy Learning Design Roadmap
TS: Translational simulation
IPO: Input-process-output
COVID19: Coronavirus disease 2019
AAPP: Accelerated Anaesthetic Practitioner Programme
NETP: National Endoscopy Training Programme
JAG: Joint Advisory Group on Gastrointestinal Endoscopy
VR: Virtual reality
