# Supplementary material for: Utilising simulation to address national workforce and quality priorities: learning from the first four years of the NHS Scotland Academy
Source: Adv Simul (Lond). 2026 Jul 14;11:71. doi: 10.1186/s41077-026-00466-3 (PMC13647756; doi:10.1186/s41077-026-00466-3)
Supplement: Supplementary file 1 [file 41077_2026_466_MOESM1_ESM.docx]

**Supplementary Table 1: Contextual detail for the two exemplar programmes**

| **Feature** | **Accelerated Anaesthetic Practitioner Programme (AAPP)** | **National Endoscopy Training Programme (NETP)** |
| --- | --- | --- |
| Workforce / quality priority | Prolonged anaesthetic practitioner training, variable access to high-impact clinical exposure and need to support elective surgical capacity | Need to improve endoscopy quality, expand training capacity, support accreditation and reduce variation in endoscopy practice across Scotland |
| Participants | Anaesthetic practitioners undertaking accelerated development towards completion of anaesthetic practitioner competencies, supported by workplace supervisors and employing Health Boards | Endoscopy practitioners, including colonoscopists and upper gastrointestinal endoscopists, with associated trainer-development and whole-team elements |
| Design and delivery contributors | NHSSA programme team, anaesthetic clinical experts, simulation educators, educational governance leads, workplace supervisors, Board representatives and digital support | NHSSA programme team, endoscopy clinical leads, national faculty, JAG-aligned trainers, regional training centres, service stakeholders and accreditation and patient partners |
| Simulation / educational modalities | Blended programme incorporating digital preparation, simulation-enabled workshops, procedural/technical skills rehearsal, scenario-based learning, feedback and workplace-based competency progression | Structured technical skills training, simulation-supported endoscopy training, virtual-reality simulation, Train the Trainer activity, best-practice courses and non-technical/behavioural skills development |
| Locations | NHSSA facilities and/or simulation-enabled educational settings, with translation into learners’ employing Boards through workplace practice, supervision and competency evidence | National and regional endoscopy training infrastructure, including dedicated training-centre activity and regional delivery across Scottish endoscopy services |
| Resources used | Simulation facilities, task trainers or procedural equipment, digital learning resources, competency frameworks, faculty time, workplace supervision and programme governance structures | Endoscopy training facilities, virtual-reality simulators, JAG-aligned course materials, best-practice consensus documents, digital learning resources, national faculty, regional training capacity and accreditation-support processes |
| Feedback and quality mechanisms | Learner and faculty feedback, supervisor and Board feedback, curriculum progression data, annual programme review, risk/action logs and programme governance review | Learner, faculty and service feedback, performance and training data, stakeholder review, accreditation-related data, annual programme review and programme governance processes |
| Translational link | Simulation-supported education was embedded within a national workforce strategy to accelerate service readiness and support anaesthetic workforce capacity | Simulation and structured training were embedded within a national strategy to improve endoscopy quality, build training capacity, support accreditation and standardise practice |
